# Retroperitoneal lymph node dissection (RPLND) for malignant phenotype Leydig cell tumours of the testis: a 10-year experience

**DOI:** 10.1186/s40064-014-0781-x

**Published:** 2015-01-14

**Authors:** Jane Hendry, Sioban Fraser, Jeff White, Prabhakar Rajan, David S Hendry

**Affiliations:** Department of Urology, Gartnavel General Hospital, G12 0YN 1053 Great Western Road, Glasgow, UK; Department of Pathology, Southern General Hospital, G51 4TF 1345 Govan Rd, Glasgow, UK; Beatson West of Scotland Cancer Centre, G12 0YN 1053 Great Western Road, Glasgow, UK

**Keywords:** Testicular cancer, Leydig cell tumour, Retroperitoneal lymph node dissection (RPLND)

## Abstract

Retroperitoneal lymph node dissection (RPLND) is a prognostic, palliative, and potentially therapeutic procedure for patients with malignant phenotype Leydig cell tumours of the testis. We reviewed the records of patients diagnosed with malignant phenotype Leydig cell tumours of the testis treated by RPLND. Modified template dissection was performed in all cases with extra-template excision of tumour mass in Stage II disease. Routine clinico-radiological follow-up was performed. Six open RPLNDs (1 re-do procedure) were performed on 5 patients diagnosed with Stage I (n = 3) and Stage II (n = 2) malignant phenotype Leydig cell tumour of the testis. Median age = 63 years (range = 55-72). Median peri-operative blood loss = 1500 ml (range = 500-1500 ml). Median operating time = 6 h (range = 4.5-6.5). Two patients with Stage II disease developed post-operative complications of acute kidney injury (n = 1) and pneumonia (n = 1). Median length of stay was 8 days (range = 6-11). RPLND specimens from patients with Stage I were tumour-free, whilst patients with Stage II disease had evidence of metastatic tumour. At latest follow-up (median = 13 months, range = 7-22), no patient with Stage I disease had radiological evidence of recurrence, however the two patients with Stage II disease had died due to tumour recurrence at 13 months and 36 months. RPLND for malignant phenotype Leydig cell testicular tumours appears to be well tolerated. Despite surgery, overall outcomes for Stage II appear to be poor due to the disease phenotype. Larger prospective multi-centre studies are required to determine the definitive criteria for surgery in Stage I disease.

**Electronic supplementary material:**

The online version of this article (doi:10.1186/s40064-014-0781-x) contains supplementary material, which is available to authorized users.

## Background

Testicular cancer is a relatively rare malignancy with ~2200 new cases diagnosed each year in the UK (Testicular cancer incidence statistics [2014]). In Scotland, ~200 new cases are diagnosed each year with a 93.4% overall crude 5-year survival rate (Summarised cancer mortality, male genital organs [2014]), largely as a result of contemporary chemotherapy strategies. Germ cell tumours represent the commonest histological subtype, and rarer variants include sex cord/gonadal stromal tumours and non-specific stromal tumours with both benign and malignant disease phenotypes (Albers et al. [2011]).

Leydig cell tumours are gonadal stromal tumours that represent less than 5% of adult testicular tumours, however are the most common non-germ cell testicular tumour (Cheville [1999]). Whilst the majority have benign clinical course, and can be fully treated by local excision or radical inguinal orchidectomy, arguably 10% are recognized to have a malignant phenotype with metastatic potential based upon the presence of specific histopathological features (Albers et al. [2011]) (Table 1). Similar to germ cell tumours, the route of spread is haematogenous and lymphatic to the retroperitoneal lymph nodes, but unlike germ cell tumours there is relative lack of sensitivity to radiotherapy and chemotherapy agents (Farkas et al. [2000]).

**Table 1:** **Histopathological features suggesting a malignant phenotype of a testicular Leydig cell tumour** (Albers P, Albrecht W, Algaba F, Bokemeyer C, Cohn-Cedermark G, Fizazi K, Horwich A, Laguna MP, European Association of U [2011])

Number	Features
I.	Large Tumour size (>5 cm)
II.	Cytological atypia
III.	Increased mitotic activity (>3 per 10 high power field [HPF])
IV.	Increased MIB-1 expression
V.	Necrosis
VI.	Vascular invasion
VII.	Infiltrative margins
VIII.	Extension beyond the testicular parenchyma
IX.	DNA aneuploidy

Retroperitoneal lymph node dissection (RPLND) can be performed subsequent to orchidectomy in patients with both organ-confined and metastatic disease, and serves as a prognostic, palliative, and potentially therapeutic procedure. The outcomes of RPLND for malignant Leydig cell tumours of the testis have been limited to a few case series (Silberstein et al. [2014]; Di Tonno et al. [2009]; Mosharafa et al. [2003]; Peschel et al. [2003]). Here, we present our national Scottish experience of RPLND for malignant phenotype Leydig cell tumours of the testis.

### Patients and methods

Between 2004 and 2014, all males within Scotland diagnosed with malignant phenotype Leydig cell tumours of the testis deemed suitable for surgery were referred to our specialist centre for consideration of open RPLND performed by a single surgeon. Diagnosis, determination of disease phenotype, and suitability for major surgery was made in local referring centres by histopathological assessment of the primary orchidectomy specimen as per European and UK guidelines (Albers et al. [2011]; Standards and datasets for reporting cancers. Dataset for the histological reporting of testicular neoplasm [2014]) prior to referral to our centre. All patients were reviewed clinically to verify suitability for surgery and primary orchidectomy specimens were subjected to centralised histopathology review to confirm histological features. Clinico-pathological parameters were recorded centrally including patient demographics, symptoms, tumour markers (α-fetoprotein protein [α-FP], β-human chorionic gonadotrophin [β-hCG], lactate dehydrogenase [LDH]), histopathology, and radiological staging by computed tomography (CT) and, where available, positron emission tomography CT (PET-CT). Clinical stage, as per TNM created by the American Joint Committee on Cancer (AJCC) was recorded based on preoperative clinical staging (Testis [2010]). Following case review by the specialist testis multi-disciplinary team, patients were offered RPLND. Pre-operative sperm banking was offered in local referring centres as per National Guidelines (SIGN [2011]). For all patients, open RPLND with modified unilateral template dissection was performed, as previously described by the Indiana group (Donohue et al. [1982]), which involves removal of lymphatic tissue from the right paracaval, precaval, and interaortocaval areas for right-sided tumours and left para-aortic and pre-aortic areas for left-sided tumours. Additional extra-template excision of tumour mass in Stage II disease was also performed where indicated by pre-operative imaging. Where possible, nerve-sparing procedures was performed to preserve ejaculatory function. Subsequent follow-up was undertaken in local referring centres and comprised CT imaging, serum tumor markers and clinical examination on an initial 3–6 monthly schedule.

## Results

Between 2004 and 2014, we performed 6 RPLNDs on 5 patients (including 1 re-do procedure) diagnosed with Stage I (n = 3) and Stage II (n = 2) malignant phenotype Leydig cell tumour of the testis. The median age at diagnosis was 63 years (range = 55-72), and there were 4 right-sided and 2 left-sided modified template RPLNDs performed. Clinico-pathological characteristics of the patient cohort are given in Table 2. Pre-operative testicular tumour markers were found to be within normal limits in all cases. No patient had any endocrine symptoms, therefore pre-operative biochemical assessment was not performed. Of the patients with Stage II disease (n = 2), only 1 patient had pre-operative chemotherapy (4 cycles of Bleomycin, Etoposide and Cisplatin), but had residual retroperitoneal disease on post-treatment radiological imaging signifying a poor treatment response. No patient with Stage I disease was subjected to pre-operative chemotherapy.

**Table 2:** **Peri-operative patient characteristics**

Case	Age [Side]	Path. features from Table1(Total)	Tumour markers	Pre-op. stage	Blood loss (ml)	Op. time (hours)	Complications	LOS (days)	Pathology at RPLND	Recurrence	DFS (months)
**1**	(63) Right	I, II, III, VI, VIII (5)	aFP 2bHCG<2 LDH 183	IIA (Ipsilateral Paraortic nodal uptake)	1500	6	Nil	11	LCT (180x65x25mm) Involvement of right ureter-> nephrectomy	Yes	N/A (died at 13 months)
**2**	(55) Left	II, III, V (3)	aFP 3bHCG<2 LDH 210	I	500	6	Nil	8	Benign	No	7 months
**3**	(72) Left	I, II, V, VI (4)	aFP 4bHCG<3 LDH 214	IIB (Ipsilateral Paraortic nodes, up to 5cm)	1500	6.5	AKI, Dialysed	8	LCT (120x85x40mm)	Yes-16 months (required further resection)	16 months (died at 36 months)
**4**	(67) Right	I, III (2)	aFP 6bHCG<3 LDH 186	I	1500	5.5	No	6	Benign	No	23 months
**5**	(61) Right	II (1)	aFP 3bHCG<3LDH 222	I	800	4.5	Pneumonia	9	Benign	No	16 months

Path = histopathological; α-FP = α-fetoprotein protein; β-hCG = β-human chorionic gonadotrophin; LDH = lactate dehydrogenase; op = operative; LOS = length of stay; LCT = leydig cell tumour; DFS = disease-free survival.

Although pre-operative ejaculatory function data was not available, a standard modified unilateral template dissection was performed for all cases of Stage I disease with attempted nerve-sparing to preserve post-ganglionic sympathetic fibres for ejaculatory function. For Stage II, an extended dissection was performed to completely resect the tumour mass as indicated by pre-operative imaging. Peri-operative details are given in Table 2, however there was no major peri-operative vascular or visceral injury. Median peri-operative blood loss was 1500 ml (range = 500-1500 ml) and median operating time 6 hours (range = 4.5-6.5).

One patient with Stage II disease (Case 1) had extensive involvement of the right ureter in tumour mass and therefore nephrectomy was performed. One patient with Stage II disease developed post-operative complications of acute kidney injury (Case 3) and one patient with Stage I disease developed pneumonia (Case 5). The median length of stay was 8 days (range = 6-11). On histopathological assessment, resected specimens from patients with Stage I were tumour-free, whilst patients with Stage II disease had evidence of metastatic tumour in the RPLND specimens. Unfortunately, post-operative ejaculatory function was not available for all patients.

**Figure 1:**
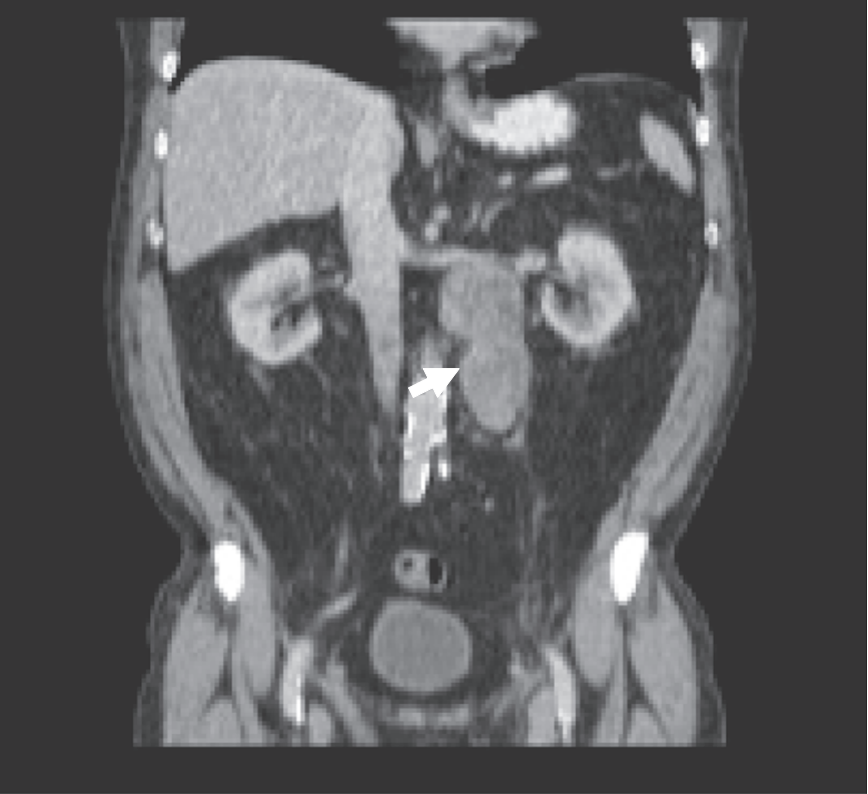
**CT scan demonstrating retro-peritoneal tumour.** Abdominal CT scan from Case 3 highlighting retroperitoneal mass on ipsilateral side to original primary tumour. RPLND yielded a 120x85x40mm mass which was histologically confirmed as metastatic Leydig cell tumour.

**Figure 2:**
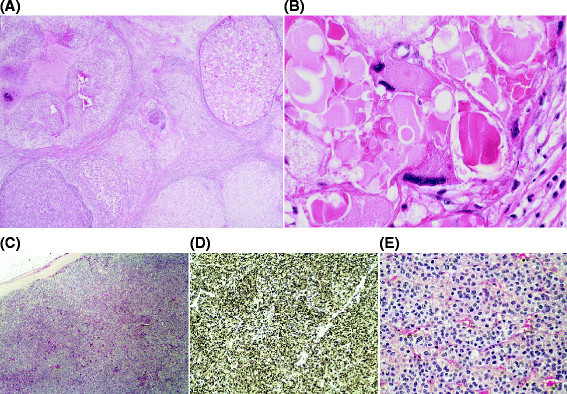
**Histopathology slides of primary and metastatic Leydig cell tumour.** Sample obtained from Case 2. **(A)** Haematoxylin and Eosin (H&E) image demonstrating tumour in orchidectomy specimen (×1.25 magnification). **(B)** H&E image demonstrating severe cytological atypia in testicular primary (×20 magnification. **(C)** H&E image demonstrating tumour in mass from RPLND (×200 magnification). **(D)** Immunohistochemical staining of RPLND specimen demonstrating expression of Inhibin (×40 magnification). **(E)** H&E image demonstrating tumour RPLND specimen (×20 magnification).

At latest follow-up (median = 13 months, range = 7-22), no patient with Stage I disease had radiological evidence of recurrence, however patients with Stage II disease had died due to tumour recurrence at 13 months and 36 months. One of the patients with Stage II disease (Case 3) developed an early left-sided pelvic recurrence adjacent to the mesentery of this sigmoid colon and ureter, and required re-resection with ureteric reconstruction with a Boari flap at 16 months post primary surgery. Figures 1 and 2 demonstrates this patient’s initial CT scan, as well as histopathology of the primary testicular tumour and resected metastases from the initial RPLND.

## Discussion

Malignant phenotype Leydig cell tumours typically present in older men with a reported median age of 62.1 (range 39–70) years (Gonzalez et al. [2007]), which is similar to our cohort. The normal endocrine function of the Leydig cell may result in elevated serum and urine sex hormone levels during tumourigenesis however, the clinical symptoms related to hormone excess are less common. Signs and symptoms of hormone excess may precede identification of a testicular mass, and in younger patients precocious puberty may be the initial symptom, with gynaecomastia being a clinical sign in older patients (Gana et al. [1995]; Ozyavuz et al. [1993]).

Leydig cell tumours are historically quoted as having 10% risk of malignant phenotype with metastatic potential (Cheville et al. [1998]; Kim et al. [1985]). An increase in the number of incidental focal testicular lesions, identified by wider use of scrotal ultrasonography, of which the majority are benign (Carmignani et al. [2003]), has led to the suggestion that metastatic rates of Leydig cell tumours may be less than traditionally thought. Hence, organ-sparing surgery by local excision in young patients with low risk disease is a potential therapeutic option (Heer et al. [2010]; Loeser et al. [2009]). However, the confident pre-operative prediction of a benign phenotype is challenging based upon current diagnostic modalities.

Given the tumour’s overall rarity and low malignant potential, and refractory response to radiotherapy and chemotherapy, there is still controversy over optimal treatment. One potential reason for this is the lack of unequivocal histopathological criteria for a malignant phenotype. Although the only universally-recognized hallmark of a malignant phenotype is the presence of metastasis, there are 9 overall features of the primary tumour thought to be indicative (Table 1), although not all of these needs to be present in tumours that metastasize (Grem et al. [1986]). In our series, all patients with histopathologically-confirmed Stage II disease had only 4 or 5 of these criteria. However, in a series (n = 29) of young (median = 43 years) patients who underwent primary surgery alone, only five patients expressed any criteria, of which only two expressed two features (Loeser et al. [2009]). Furthermore, no metastases were observed over a median 49-month follow-up period.

The development of metastases typically occurs within 2 years of the initial diagnosis, although has been reported up to 8 years after orchidectomy (Bertram et al. [1991]). Metastases have similar histological features to the original tumour (spreading to retroperitoneal lymph nodes (70%), liver (45%), lung (40%) and bone (25%), and are particularly chemo-radio-resistant, with radiation only effective in some cases to palliate pain symptoms (Bertram et al. [1991]). Hence RPLND may have prognostic, palliative, and potentially therapeutic application in at risk patients with Stage I, as well as Stage II disease.

Studies reporting the outcome of RPLND for malignant phenotype Leydig cell tumours are limited (Silberstein et al. [2014]; Di Tonno et al. [2009]; Mosharafa et al. [2003]; Peschel et al. [2003]). Mosharfa *et al.* report outcomes of RPLND (n = 17) for sex cord stromal tumours, including malignant phenotype Leydig cell tumours (n = 6) (Mosharafa et al. [2003]). Three cases had Stage I disease had were disease-free at latest follow-up (range = 25-135 months), and three cases had Stage II disease at RPLND, and subsequently died (at range = 11-52 months) despite further treatment (surgery, chemo- or radio-therapy). The latter patients had initially presented with Stage I disease and were managed by surveillance, highlighting the potential benefit of early RPLND.

Di Tonno *et al.* report outcomes of RPLND (n = 5) undertaken for Stage I (n = 3) and Stage II (n = 2) malignant phenotype Leydig cell tumours (Di Tonno et al. [2009]). During follow-up (range = 24-214 months), there were no recurrences reported. The median age of this cohort was low (mean = 36 years), and since age is a poor prognostic factor for Leydig cell tumours (Albers et al. [2011]), one might expect a lower rate of recurrence and metastatic disease compared with our own patient cohort. There is only one study of minimally-invasive (laparoscopic) RPLND for patients with Stage I malignant phenotype Leydig cell tumours (n = 6) with a mean age of 41 years (range 29–58) (Peschel et al. [2003]). However, there were two intra-operative vascular complications requiring open conversion. During a mean follow-up of 12 months (range 3–29 months), no recurrences were identified.

The most recent series is from Memorial Sloan Kettering Cancer Centre: Of the 48 patients with sex cord stromal tumours described, 6 patients with either Stage I (n = 4) or Stage II (n = 2) malignant phenotype Leydig cell tumours were managed by RPLND. Interestingly, two patients with Stage I disease, who harboured 5 pathological features (Table 2), developed early relapse and died of metastatic disease within 24 months of surgery (of which one had positive nodal disease), whilst the other 2 remained disease-free. Of the patients with Stage II disease, one had recurred but was still alive at 49 months after surgery. The median age of the RPLND-managed cohort was 48 years (interquartile range = 37-53), and median follow-up time was 68 months (range = 49-173).

We present the largest UK series of RPLND for malignant phenotype Leydig cell tumours of the testis. Although limited by a small patient cohort with a short follow-up period and a lack of functional outcomes, our experience suggests that RPLND is well tolerated with minimal toxicity and good disease-free outcomes in Stage I disease. Our operating time (median = 6 h, range = 4.5-6.5) and peri-operative blood loss (median = 1500 ml, range = 500-1500 ml) appeared greater than the parameters from the only report including these outcomes (mean time = 190 mins, range = 150-225, and mean estimated blood loss = 110 ml, range = 20-350), but may be due to the minimally-invasive technique employed (Peschel et al. [2003]). A further limitation of our report is the absence of information on patients managed in local centres by surveillance and/or chemotherapy.

Taken together with previously published data, there is still insufficient evidence to recommend RPLND as standard of care for all patients. For young patients with very few (≤1) malignant histological features, a period of active monitoring may be considered. However, for older patients, those with a larger number of malignant histological features, or those with Stage II disease, RPLND may, at least, offer good palliation and a possibility of cure. Larger prospective multi-centre studies, with centralized histopathological reporting, are required to determine the definitive role for surgery in Stage I disease.

### Ethical standards

The manuscript does not contain any experimental clinical studies or identifiable patient data.

## Authors’ original submitted files for images

Below are the links to the authors’ original submitted files for images. Authors’ original file for figure 1 Authors’ original file for figure 2
