# Supplementary figures and images for: Retroperitoneal lymph node dissection (RPLND) for malignant phenotype Leydig cell tumours of the testis: a 10-year experience
Source: Springerplus. 2015 Jan 14;4(1):20. doi: 10.1186/s40064-014-0781-x (PMC4300307; doi:10.1186/s40064-014-0781-x)

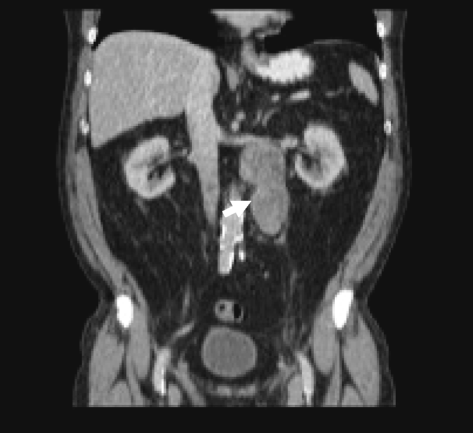

Supplement: Supplementary file 1 — Authors’ original file for figure 1 [file 40064_2014_781_MOESM1_ESM.gif]

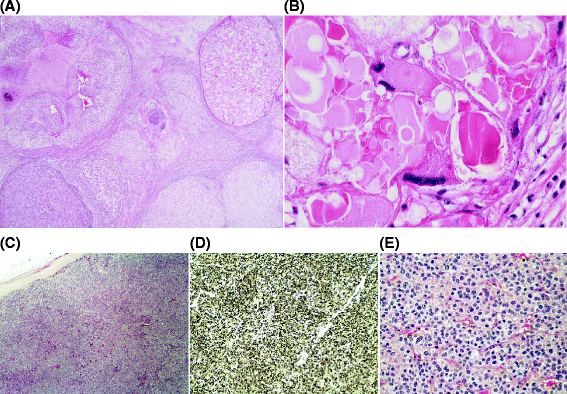

Supplement: Supplementary file 2 — Authors’ original file for figure 2 [file 40064_2014_781_MOESM2_ESM.gif]
